# Dynamic Modification of Fermi Energy in Single-Layer Graphene by Photoinduced Electron Transfer from Carbon Dots

**DOI:** 10.3390/nano10030528

**Published:** 2020-03-15

**Authors:** Angelo Armano, Gianpiero Buscarino, Fabrizio Messina, Alice Sciortino, Marco Cannas, Franco Mario Gelardi, Filippo Giannazzo, Emanuela Schilirò, Simonpietro Agnello

**Affiliations:** 1 Dipartimento di Fisica e Chimica-Emilio Segrè, Università degli Studi di Palermo, Via Archirafi 36, 90123 Palermo, Italy; angelo.armano@unipa.it (A.A.); gianpiero.buscarino@unipa.it (G.B.); fabrizio.messina@unipa.it (F.M.); alice.sciortino02@unipa.it (A.S.); marco.cannas@unipa.it (M.C.); franco.gelardi@unipa.it (F.M.G.); 2 Dipartimento di Fisica e Astronomia-Ettore Majorana, Università degli Studi di Catania, Via Santa Sofia 64, 95123 Catania, Italy; 3 ATeN Center, Università degli Studi di Palermo, Viale delle Scienze, Edificio 18, 90128 Palermo, Italy; 4 Consiglio Nazionale delle Ricerche-Istituto per la Microelettronica e Microsistemi, Strada VIII 5, 95121 Catania, Italy; filippo.giannazzo@imm.cnr.it (F.G.); emanuela.schiliro@imm.cnr.it (E.S.)

**Keywords:** graphene, nanomaterial, 2D material, carbon, Raman spectroscopy, material science

## Abstract

Graphene (Gr)—a single layer of two-dimensional sp^2^ carbon atoms—and Carbon Dots (CDs)—a novel class of carbon nanoparticles—are two outstanding nanomaterials, renowned for their peculiar properties: Gr for its excellent charge-transport, and CDs for their impressive emission properties. Such features, coupled with a strong sensitivity to the environment, originate the interest in bringing together these two nanomaterials in order to combine their complementary properties. In this work, the investigation of a solid-phase composite of CDs deposited on Gr is reported. The CD emission efficiency is reduced by the contact of Gr. At the same time, the Raman analysis of Gr demonstrates the increase of Fermi energy when it is in contact with CDs under certain conditions. The interaction between CDs and Gr is modeled in terms of an electron-transfer from photoexcited CDs to Gr, wherein an electron is first transferred from the carbon core to the surface states of CDs, and from there to Gr. There, the accumulated electrons determine a dynamical n-doping effect modulated by photoexcitation. The CD–graphene interaction unveiled herein is a step forward in the understanding of the mutual influence between carbon-based nanomaterials, with potential prospects in light conversion applications.

## 1. Introduction

Nanomaterials have rapidly attracted the interest of frontiers research in material science [1]. In particular, among the many varieties of nanostructured systems, low-dimensional materials and carbon-based nanomaterials play an important role for research in nanotechnology [2]. Graphene (Gr), a single layer of carbon atoms in the hexagonal honeycomb structure of a single layer of graphite, was the first two-dimensional material to be isolated and investigated [3]. Beyond its fascinating monoatomic thickness, its low-dimensionality gives Gr peculiar properties—high charge carrier mobility, high thermal conduction, good optical transparency, chemical stability, mechanical resistance, and flexibility [4,5,6]—which justify the intensive research on it, in view of applications in microelectronics: field effect transistors, radio-frequency transistors, vertical THz transistors [7,8,9], volatile memories [10], composite nanomaterials [11,12,13], solar cells, and rigid or flexible capacitive systems [4,14,15,16,17]. The large surface area of Gr and the presence of delocalized electrons result in a strong interaction between Gr and surrounding species which allows charge–exchange related processes, such as doping processes involving molecular adsorption [18,19] and charge transfer to/from other nanosystems [13,20,21,22,23]. In regard to these Gr composites, most of literature works concern the interaction of graphene with metal nanoparticles [24,25,26,27], transition metal oxides [28,29] or transition metal calchogenide quantum dots [12], and graphene oxide—mainly investigated in liquid phase. Despite that such composite nanomaterials are of cross-disciplinary interest for application in photocatalysis, molecular detection, and microelectronics, most of them are hindered by severe practical limitations, such as lack in visible emission, toxicity of materials, and inapplicability in solid-state microelectronics.

In recent preliminary studies, we have explored a new type of all-carbon nanocomposite, in order to overcome the above cited restrictions [30,31]. In particular, Gr fabricated by chemical vapor deposition (CVD) has been selected for its high conductive properties and good implementation for solid state devices [32]. On the other hand, carbon dots (CDs), a class of carbon-based and nontoxic nanoparticles have been used [33]. The high efficiency and the strong sensitivity to the environment of CDs photoluminescence (PL) stimulates the interest on the tailoring of CDs with the purpose to substitute the more common, but toxic, metal-calchogenide quantum dots [34,35,36]. Despite their nanometric size, such nanoparticles cannot be considered 0D materials since they do not show quantum confinement effects. More specifically, the photocycle of CDs involves both their carbon core and their surface passivation groups [37], and the consequent exposure of negative charge at the CDs surface enables charge transfer mechanisms with other close ions [38,39,40,41] or molecular species [42,43]. We proposed a similar process in order to describe the interaction between CDs and Gr supported on $\mathrm{Si}O_{2}$ in a solid composite, wherein the CD emission efficiency was affected by the surface of deposition [30], noting also the lack of structural modification of Gr decorated with CDs [31] and the stability of CDs to soft thermal treatment [30]. Based on the above considerations, the integration of such a composite system in microelectronic devices would present several advantages compared to other solution: inexpensive and nontoxic nanoparticles as optically active layer, and CVD Gr—which allow a better implementation in solid state technology compared to other synthesis of this nanomaterial—as conductive layer.

In the present work, we deepen the interaction between CDs and Gr by the simultaneous investigation of the electronic properties of both materials and the role of graphene doping. In particular, the emission efficiency of CDs is found to be strongly reduced when they are in contact with Gr compared to CDs in contact with ${\mathrm{SiO}}_{2}$. At the same time, changes in the Raman spectra of Gr, induced by the presence of photoexcited CDs, indicate a rising in the Fermi energy compared to Gr without CDs deposited on. On the contrary, no modification in Fermi energy of Gr is found when the CDs are kept in their unexcited state. In order to explain these findings, we propose a model involving a photoinduced electron transfer (PET) process from CDs to Gr. The surface electron of CDs migrate to Gr thanks to the close contact and the favorable energy alignment of the surface orbitals of the two nanomaterials. Thus, because of the modification of the charge carriers concentration of Gr, CDs can be used as a photo-activated source of n-doping or electron injection.

## 2. Materials and Methods

### 2.1. Graphene (Gr)

Commercial monolayer Gr produced by Graphenea Inc. was used. According to the manufacturing specification, Gr was grown on Cu foil by chemical vapor deposition technique at a temperature of 1000 °C, using ${\mathrm{CH}}_{4}$ as carbon source. In order to transfer the Gr on the substrates, a layer of Poly(methyl methacrylate) (PMMA) was spin coated onto Gr/Cu, then a thermal release tape (TRT) was laminated on PMMA. The Cu foil was etched by immersion in an ammonium persulfate water solution. The remaining TRT/PMMA/Gr stack was transferred to the substrate by thermocompression printing causing the release of TRT, and was cleaned from PMMA layer by an acetone bath. Standard silicon substrate with a surface covered by 300 nm of ${\mathrm{SiO}}_{2}$ was used, thus obtaining a Gr/${\mathrm{SiO}}_{2}$/Si wafer (in the following named Gr/${\mathrm{SiO}}_{2}$). Reference data for CDs-free Gr are reported from Reference [44].

### 2.2. Carbon Dots (CDs)

CDs were synthesized using citric acid monohydrate, as carbon precursor, and urea as nitrogen precursor, both dissolved in an aqueous solution. The carbonization process of the solution was performed by microwave irradiation until the complete evaporation of water occurred [45,46]. In previous characterization, these carbon nanoparticles resulted to have typical size values in the range 1–10 nm [45]. From the resulting CDs powder, a suspension in ethanol was prepared, with a concentration of CDs equal to 0.1 g/L. No purification of CD solution has been applied. For the study of CDs-Gr composite in solid phase, 1 µL volume of the CDs solution was deposited by drop-casting technique. The depositions were performed in fume hood in order to accelerate the drying of the solvents.

### 2.3. Thermal Processing

The Gr was doped by thermal treatment in a controlled atmosphere. In particular, the treatment was performed in a stainless steel chamber of about 100 mL volume with controllable temperature and pressure at temperature equal to 300 °C for 2 h. After a prevacuum at pressure of 0.5 mbar, the oxygen gas ($O_{2}$) was injected in the chamber, at pressure of 2 bar with 20 ppm mol of impurities.

### 2.4. Micro-Raman (μ-Raman) and Microphotoluminescence (μ-PL) Spectroscopy

μ-Raman and μ-PL spectroscopy by 532 nm (2.33 eV) excitation laser were performed by using SENTERRA μ-Raman spectrometer (Bruker, Europe) equipped with a confocal optical microscopy system with 50× optical magnification, a best spectral resolution equal to 9 cm^−1^, and a data pitch equal to 0.5 cm^−1^. All the measurements were performed with nominal power equal to 5 mW. Target area of measurement (about 1 × 1 μm^2^) was defined by the size of focused laser spot and imposed by diffraction limit resolution of optics. μ-Raman Spectroscopy by 633 nm (1.96 eV) excitation laser was performed using a LabRam HR-Evolution spectrometer (Horiba, Europe) equipped with a confocal optical microscopy system with 100× optical magnification, a best spectral resolution equal to 7 cm^−1^, and a data pitch equal to 1 cm^−1^. All the measurements were performed with at nominal power equal to 5 mW on a target area of 1 × 1 μm^2^. All the spectra were aligned to the silicon band located at 520.7 cm^−1^ [47,48]. For each sample, a set of at least 20 measurements was acquired in order to evaluate heterogeneity and to determine the mean values for the Gr spectroscopic features. G and 2D band peak positions, ν_G_ and ν_2D_ respectively, were extracted from each Raman spectrum of Gr [49]. The uncertainty of the reported values is expressed in terms of one standard deviation of each values’ distribution.

Correlation analysis G and 2D peak positions (G-2D graph) was used to disentangle strain and charge doping of Gr [49]. Concerning the spectra acquired with 532 nm (2.33 eV) laser, for the calculation of doping, the same approximated relation of Reference [50] was used. For the calculation of strain, the dispersion relation that binds volume and mode frequencies and a Grüneisen parameter for graphene equal to 3.55 were used [51,52,53]. The reference axes for strain and doping according to the excitation energy of 2.33 eV are marked in G-2D graphs by two lines whose slopes are equal to 2.45 and 0.7, respectively, and define the pairs of values (ν_G_, ν_2D_) equivalent to only strain or p-type doping effects [50,54]. The intersection between the two axes at (1582 cm^−1^, 2670 cm^−1^) determines the ideal configuration for unstressed and undoped Gr. Other positions in the graph are interpreted in terms of the vectorial compositions of concurrent doping and strain effects whose single evaluation is achieved by the projection of each point on the respective axes [51,52,53]. For spectra acquired with 633 nm laser, the doping and strain axes were moved according to References [55,56] by taking into consideration the phonon dispersion of Gr Raman bands [57,58] which implies the shift of Gr Raman bands by varying the laser energy. The strain and doping axes slope are equal to 2.5 and 0.7, respectively, and their intersection is placed at (1582 cm^−1^, 2634 cm^−1^). In this case, the doping of various samples was compared only qualitatively.

μ-PL mapping was performed on a target area of 25 × 25 μm^2^ by acquiring 625 measurements at 1 μm from each other, and that were therefore arranged in a 25 × 25 grid. Then, a μ-PL map was obtained by reporting point-by-point the PL intensity of CD emission. In this work, mapping image is shown after interpolation correction. Raw mapping is reported in Supporting Information (see Figure S1).

### 2.5. Liquid-Phase Optical Characterization

Steady-state absorption measurements of ethanol-dispersed CDs were performed by a double-beam spectrophotometer V-560 (JASCO, Japan) in the range of 250–750 nm in a 1 cm quartz cuvette. The emission spectrum of ethanol-dispersed CDs was acquired by a FP-6500 spectrofluorometer (JASCO, Japan) in a 1 cm cuvette with a 3 nm resolution bandwidth.

### 2.6. Atomic Force Microscopy (AFM) and Optical Microscopy (OM)

AFM measurements were performed in Tapping Mode by using a FastScan Bio (Bruker, Europe) and a FastScan A (Bruker, Europe) probe with tip radius approximately equal to 5 nm. AFM images were acquired on different micrometric scales, up to 5 × 5 μm^2^ of area. The analysis was performed by Gwyddion v. 2.52. Optical images were acquired by using the optical microscope included in Bruker SENTERRA spectrometer: Olympus BX51 reflected light microscope (Bruker, Europe) provided with video camera Infinity 1 and objective lens with 0.75 numerical aperture and 50× magnification. Postprocessing of acquired images consisted of color curves modification. Raw images are reported in Supporting Information file (see Figures S2 and S3).

## 3. Results and Discussion

### 3.1. Spectroscopic Characterization of Samples

#### 3.1.1. Dispersed and Deposited CDs

**Figure 1:**
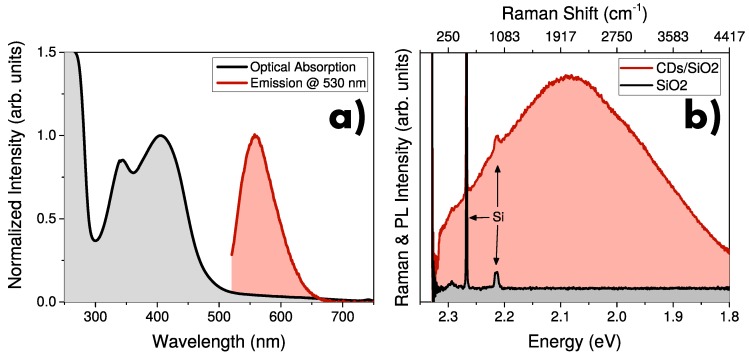
(**a**) Optical absorption (black) and photoluminescence (PL) emission excited at 530 nm (red) of ethanol-dispersed carbon dots (CDs). (**b**) μ-Raman/PL spectra of CDs deposited on ${\mathrm{SiO}}_{2}$/Si substrate (red) and bare substrate (black). Raman bands of silicon are highlighted by arrows.

Typical optical absorption and emission of CDs dispersed in liquid phase are reported in Figure 1a. As discussed in previous works by Sciortino et al., optical absorption of CDs is characterized by two main contributions: an edge below 300 nm due to band-to-band transition of the crystalline core; and a composite band between 300 and 500 nm related to transition towards midgap levels belonging to surface states. Then, absorption fades with further increase of wavelength. Moreover, CDs are characterized by a bright tunable photoluminescence, which can be excited over a broad spectral range, extending from the UV down to about 550 nm [38]. Despite that the maximum emission intensity was found for an excitation corresponding to the absorption peak at about 400 nm [38], for the aims of this work it is useful to consider the emission of CDs for higher excitation wavelengths. In the case of a green excitation at 530 nm, the PL of CDs is located at ∼ 580 nm (∼ 2.1 eV). On the other hand, no emission is found by using excitation at even lower energies.

In this work, we deposited CDs on Gr and ${\mathrm{SiO}}_{2}$ substrates in order to study their interactions with the substrate. μ-PL of CDs deposited on ${\mathrm{SiO}}_{2}$/Si substrate is reported in Figure 1b. Here, the emission spectrum of CDs is peaked at about 2.1 eV, in accordance with what was found in liquid phase [38]. It is worth noting that the narrower Raman bands of silicon substrate are superimposed to the PL of CDs (peaks at about 300, 520, and 990 cm^−1^). Such interpretation is strengthened by the evaluation of the substrate spectrum, where no stray light or undefined background are found. Furthermore, the lack of any Raman signal of CDs can be ascribed both to the low concentration of nanoparticles and to the competitive role of PL, which dominate the interaction between CDs and light. Further characterization of CD synthesis, structure, and photophysics can be found in References [38,45,46,59].

#### 3.1.2. Graphene

**Figure 2:**
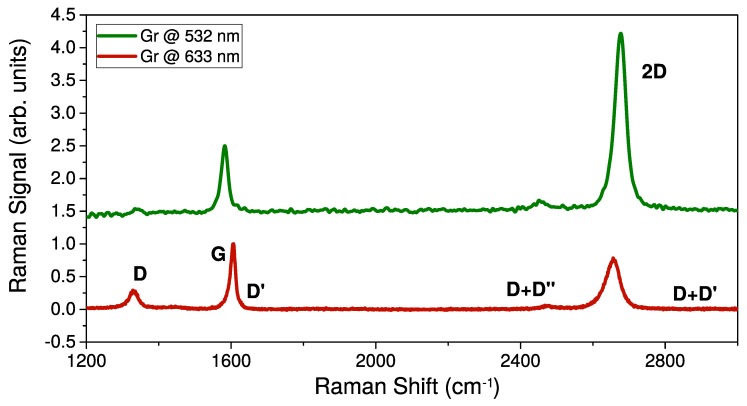
Raman spectra acquired by laser excitation at 532 nm (green) and 633 nm (red) of graphene (Gr) transferred on ${\mathrm{SiO}}_{2}$/Si. Labels indicate the characteristic bands of Gr. Both spectra are normalized on G band peak amplitude. Arbitrary vertical shift is applied.

Typical Raman spectra of Gr sample obtained by using 532 and 633 nm lasers are reported in Figure 2. Both spectra show the characteristic bands of graphene (G, 2D, D, D’, *…*) and, according to literature, many spectroscopic features are strongly influenced by the different laser excitation [57,58]: compared to silicon substrate, Gr features a different scattering efficiency upon varying laser excitation (see Figure S4); and also the intensity, width, and peak position of the characteristic Raman bands of Gr are strongly modified. By focusing attention on the spectrum acquired at 532 nm, since the intensity ratio between 2D and G band is higher than 1 we can confirm the single-layer morphology of our Gr sample [60].

Furthermore, the very low relative intensity of D band suggests that even after the transfer onto ${\mathrm{SiO}}_{2}$/Si, the defect concentration in Gr is still low. Finally, we point out that no background correction was performed on these spectra. Therefore, possible PMMA residuals onto the sample due to the transfer process are too low of a concentration to yield a detectable signal (both PL and Raman) by the experimental parameters we used.

### 3.2. CDs-Graphene Morphology

**Figure 3:**
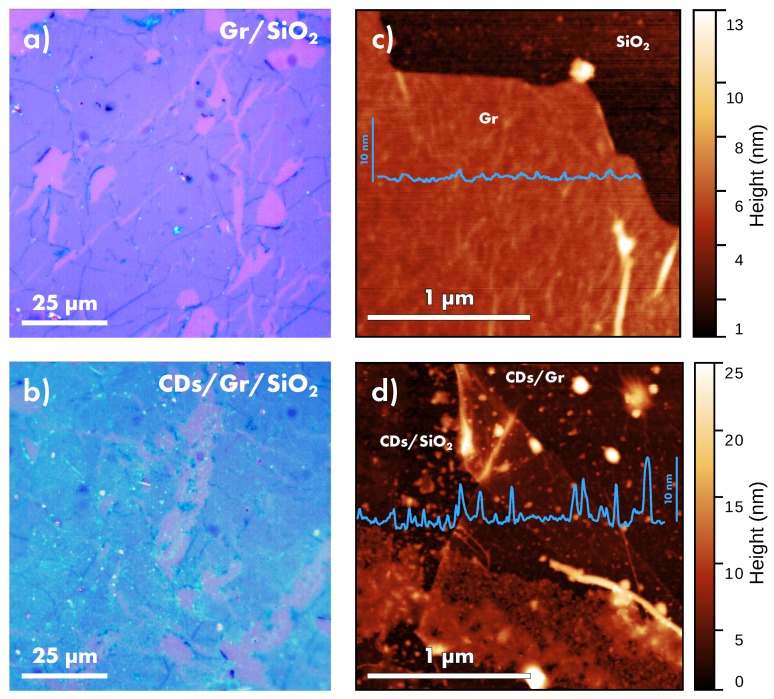
Surface morphology evaluated by (**a**,**b**) optical microscopy, and by (**c**,**d**) atomic force microscopy of Gr/${\mathrm{SiO}}_{2}$ and CDs/Gr/${\mathrm{SiO}}_{2}$, respectively. In panel (**d**), the CDs deposited on ${\mathrm{SiO}}_{2}$ (CDs/${\mathrm{SiO}}_{2}$) and CDs deposited on graphene (CDs/Gr) can be distinguished. In addition, in panels (c,d), representative profile is reported (blue line) on the corresponding section line.

The deposition of CDs on Gr/${\mathrm{SiO}}_{2}$ was performed by drop-casting technique. As shown in Figure 3a, because of many cracks in its structure, the Gr does not cover the entire surface of the ${\mathrm{SiO}}_{2}$/Si, and some parts of the ${\mathrm{SiO}}_{2}$ surface remain uncovered. As a consequence, during the deposition, the CDs are spread on the entire surface of the samples, and both the ${\mathrm{SiO}}_{2}$ and Gr surfaces are covered by CDs. Therefore, the CDs deposited on Gr/${\mathrm{SiO}}_{2}$ will be labeled as CDs/Gr and CDs/${\mathrm{SiO}}_{2}$ in the following, by distinguishing the specific surface they are in contact with. Surface morphology of CDs/Gr/${\mathrm{SiO}}_{2}$ sample is shown in Figure 3b by optical microscopy, wherein the deposition of CDs causes the appearance of a light blue halo over the sample, with no preferential spread on the surface of Gr (light blue regions) or ${\mathrm{SiO}}_{2}$ (violet regions). The spread of single CDs on the surface of Gr/${\mathrm{SiO}}_{2}$ at the microscale can be evaluated by the AFM micrographs shown in Figure 3c,d.

Therein, as emphasized by the blue section profile in Figure 3d, nanoparticles are clearly recognizable on the flat surfaces of both ${\mathrm{SiO}}_{2}$ and Gr, especially if compared to the bare surface without CDs (Figure 3c).

### 3.3. CDs-Graphene Interaction

**Figure 4:**
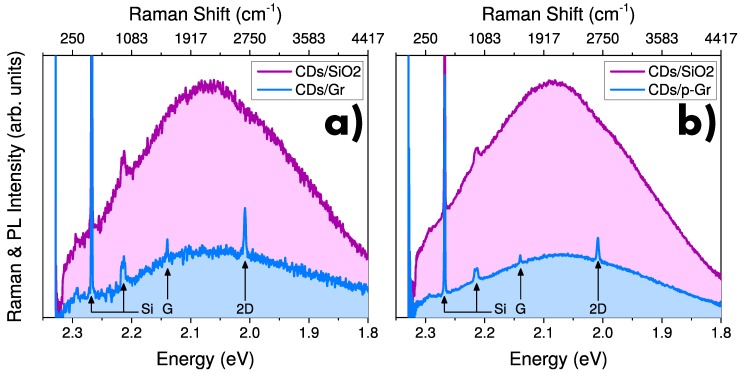
μ-PL excited at 532 nm of CDs deposited on contiguous regions of graphene and ${\mathrm{SiO}}_{2}$ for (**a**) CDs/Gr and (**b**) CDs/p-Gr. Raman bands of graphene and silicon are marked by arrows and labels. Energy axis is reported both in terms of absolute value (bottom), and in terms of the Raman shift, that is, the difference relative to the laser energy (top).

Two different kinds of CDs-graphene composites are studied. The first one, labeled CDs/Gr, was obtained by depositing CDs on native Gr, that is, the as-received sample of Gr characterized by no doping. The second sample, labeled CDs/p-Gr, was obtained by subjecting a CDs/Gr sample to thermal treatment in $O_{2}$ atmosphere. The effects of such treatments on graphene and CDs (taken separately) have already been discussed by the authors in recent works. Concerning graphene, the treatment induces a p-type doping, as indicated by the blueshift of both G and 2D Raman bands (see Figure S5). Further details about doping of graphene by thermal treatment in $O_{2}$ can be found in References [44,61,62]. Besides, when CDs are subjected to a similar thermal treatment their emission capability is preserved with minor modification [30]. In the following, the spectroscopic features of CDs/Gr and CDs/p-Gr are reported and compared. In particular, the analysis of the composite by a μ-Raman setup allows us to acquire simultaneously the Raman spectrum and the PL band emitted by CDs. Raman/PL spectra of CDs/Gr and CDs/p-Gr excited at 532 nm (2.33 eV) are shown in Figure 4 and compared to the PL of CDs deposited on the portion of substrate uncovered by graphene (that is surface ${\mathrm{SiO}}_{2}$). The spectra differ for the Raman bands superimposed to the PL of CDs: those related to silicon when CDs are in contact with ${\mathrm{SiO}}_{2}$ surface, with the addition of those of graphene when CDs are deposited on it. Most importantly, we note that the emission of CDs is influenced by the surface they are deposited on. In fact, CDs deposited onto graphene (both doped and undoped) feature a reduced emission efficiency compared to those deposited onto ${\mathrm{SiO}}_{2}$. It is important to note that such evaluation must be performed by comparing couples of measurements acquired on contiguous regions at the border of a Gr flake, aiming to reduce contribution of CD concentration in the evaluation of measured PL intensity. Despite that only two representative couples of measurements are reported here, the PL quenching effect of graphene was found true in a large number of comparisons.

**Figure 5:**
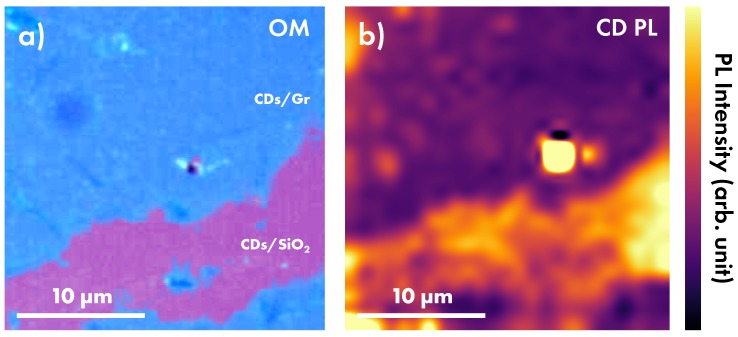
(**a**) Optical microscopy image and corresponding (**b**) μ-PL mapping excited at 532 nm of CDs deposited on contiguous regions of undoped graphene (CDs/Gr) and ${\mathrm{SiO}}_{2}$ (CDs/${\mathrm{SiO}}_{2}$). The intensity of CD emission (CD PL) is reported in the color-bar.

A more accurate evidence of graphene-induced quenching of CD PL is reported by the μ-PL mapping shown in Figure 5, where 625 measurements were acquired on a square grid in a 25 × 25 μm^2^ area where CDs were deposited onto Gr or ${\mathrm{SiO}}_{2}$. As reported in Figure 5b, different emission intensity is found across this region and it is clearly visible a strong contrast in perfect correspondence of Gr or ${\mathrm{SiO}}_{2}$ surfaces shown in Figure 5a. In particular, the emission intensity of CDs/Gr is about 30%–50% of that measured for CDs/${\mathrm{SiO}}_{2}$, thus confirming the quenching effect of graphene and suggesting the opening of a further nonradiative relaxation channel for photoexcited CDs deposited on Gr. Further heterogeneity is found on both Gr or ${\mathrm{SiO}}_{2}$ surfaces, probably due to cluster formation. However, such variability is so slight that the contrast between Gr and ${\mathrm{SiO}}_{2}$ surfaces is well visible.

**Figure 6:**
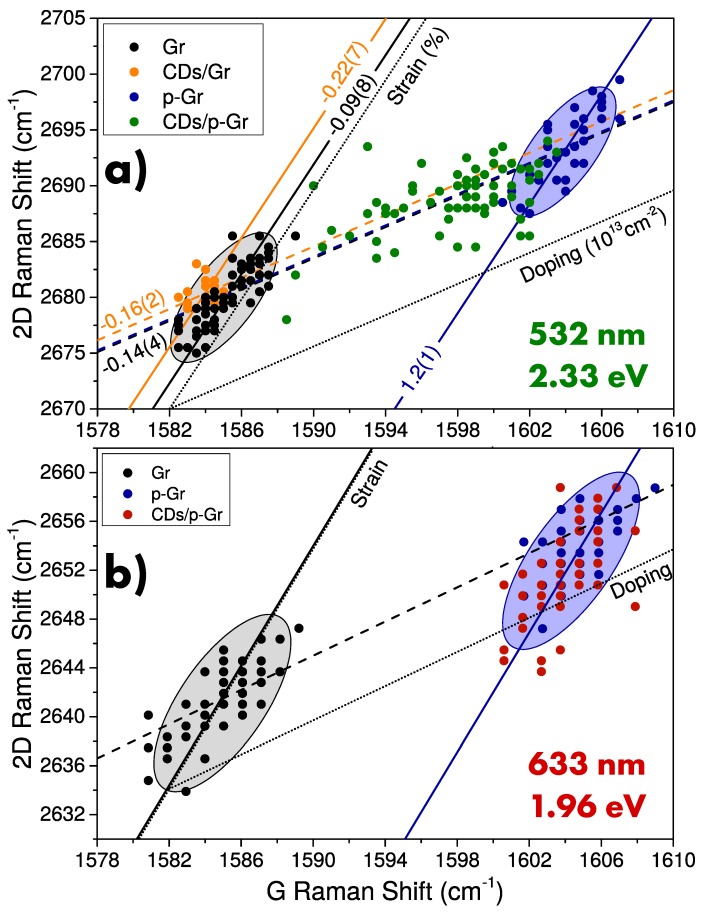
G-2D correlation graph at (**a**) 532 nm and (**b**) 633 nm laser excitation of undoped (black dots) and p-doped Gr (blue dots) (reported from Reference [44] for 532 nm only), CDs/Gr (orange dots), and CDs/p-Gr(green/red dots). Doping and strain axes are marked by dotted lines and reference levels are pointed out by continuous and dashed lines, respectively.

In order to evaluate a possible influence of CDs on Gr properties, we investigated the Raman spectrum of Gr and p-Gr with or without CDs deposited on. In particular, we studied the correlation between G and 2D bands in the calibrated G-2D graph reported in Figure 6. In fact, this is a well-established method which allows us to evaluate the tensile or compressive strain of graphene lattice, and from the electronic point of view, the charge doping of graphene that is directly correlated to the charge-carrier concentration (both for electrons and holes) and therefore to the Fermi energy [49,50]. In particular, as discussed in the Materials and Methods section, graphene strain and doping are revealed as shifts of the point cloud along two well-defined axes [49]. As shown in Figure 6a, the effects of CDs deposition are evaluated by the comparison between the point clouds of native Gr and CDs/Gr where only minor modifications are recognizable. A slight increase of compressive strain is found, suggesting a soft deformation of Gr structure as effect of nanoparticles weight. Such a modification is larger in absolute value, and opposite in sign than the effect of bare solvent deposition reported in Figure S6a, which induces a decrease of strain by softening the interaction between Gr and the substrate. On the other hand, a slight shift of CDs/Gr point cloud along the doping axis is revealed, whereas a minor modification is induced by the bare solvent. This shift can be ascribed to a minor influence of CDs on the electronic structure of Gr occurring during the collection of Raman signal. However, no quantitative estimation of the Fermi energy shift can be performed in this region of G-2D graph, where the relation between Fermi energy and spectral position of the G and 2D bands is complex and highly nonlinear [49,60].

The influence of CDs on Fermi energy of Gr can be much better investigated by using p-doped Gr rather than undoped Gr. In fact, as previously stated, the doping process blueshifts both G and 2D Raman bands (Figure S5), so as to move the respective point cloud in a region of G-2D graph where the sensitivity of this technique to differential doping is maximum [49,60]. In this case, the G-2D graph analysis for samples subjected to doping process indicates a strong difference between bare p-Gr and CDs/p-Gr. In fact, as shown in Figure 6a, the point cloud of CDs/p-Gr is placed at intermediate doping values, between bare p-Gr and native Gr point clouds, and it features a more complex shape. In particular, a larger area of G-2D graph is occupied, featuring a wide distribution of strain and doping configurations. For the latter, redshift of Raman bands indicates a competitive n-doping due to the presence of CDs. The same analysis was performed by using a laser excitation at 633 nm (1.96 eV). At this energy, the light absorption of CDs vanishes (see Figure 1) and no emission is revealed. As shown in Figure 6b, we can note that in this case, the point clouds of p-Gr and CDs/p-Gr are perfectly overlapping, thus indicating no influence of CDs on the electronic structure of Gr. Therefore, Gr seems to be affected only by photoexcited CDs. Finally, the effect of bare solvent on p-Gr for both light excitations is reported in Figure S6a,b, where a completely different modification is found, and is hence unrelated to the effect of CDs.

### 3.4. Model for CD-Gr Interaction

**Figure 7:**
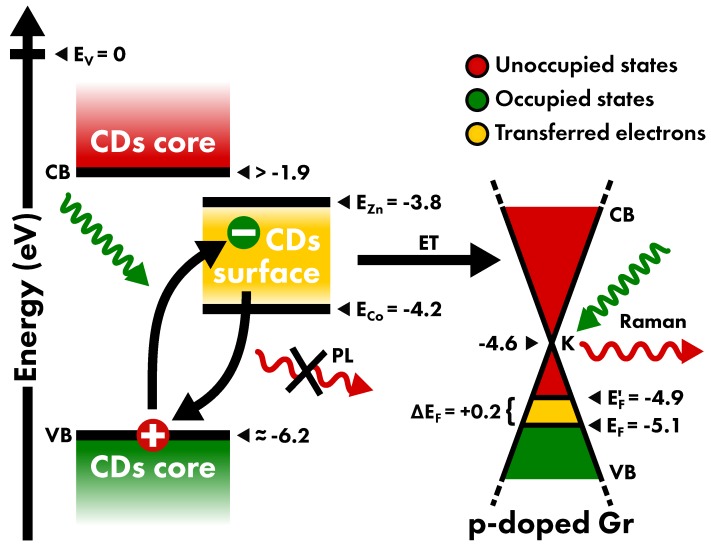
Energy levels scheme of CD core electronic bands, CD surface states, and of Gr electronic bands at K point (Dirac cones) relative to the energy of the vacuum level. The elements of the CDs-Gr interaction described in the proposed model are reported. The laser light (green arrows) is absorbed by CDs and scattered by Gr, thus generating CD PL and Gr Raman signals (red arrows), respectively. In CDs, electrons are promoted from the core valence band (VB) to surface states, for which the energy distribution is between the energies of zinc and cobalt ions. The electron migrates from the surface states towards the conduction band (CB) of Gr by electron transfer (ET). Finally, after an ultrafast relaxation towards lower energy states, the states in the VB of Gr are populated, as attested by the increase of Fermi energy revealed by Raman signal.

Based on the gathered information, and by taking into account previous investigations (References [30,38]), we propose the interpretative model of the interaction between CDs and Gr shown in Figure 7. As a consequence of laser irradiation, the used spectrometer collects both the PL of CDs and the Raman scattering of Gr. The light absorption of CDs occurring for 2.33 eV laser causes the extraction of an electron from their core towards states exposed to the surface of the nanoparticles [38]. Thus, if the energies of the involved states are favorably aligned, the close contact of CDs surface and Gr orbitals admits charge transfer between the two materials.

The energy levels scheme of the present system can be reconstructed as follows, by taking as reference the energy of vacuum level E_v_ = 0. Since the native Gr has no relevant charge doping, the Fermi level is near the K point of Gr band structure. The energy position of this level is described by the work function of undoped Gr equal to φ_Gr_ = −4.6 eV [63]. The increase of 1.2(1) × 10^13^cm^−2^ charge carriers by p-doping treatment reported in Figure 6a implies a reduction of Fermi Energy by ΔE_F_ = −0.5 eV [64,65,66], thus lowering the work function to approximately φ_p-Gr_ = −5.1 eV.

Concerning CDs, despite that no direct measurement for the electron affinity of β-C_3_N_4_ core of CDs is known, the alignment of CD electronic structure can only be hypothesized on the basis of their spectroscopic features. The energy position of the surface states is straddled by limiting values estimated based on their interactions with metal ions [67]. This effect has been related to the redox potential, which measures the tendency of a chemical species to accept electrons. The most representative cases are for zinc and cobalt ions, which are characterized by redox potential equal to V_$Zn^{2+}$_ = −0.76 V and V_$Co^{2+}$_ = −0.28 V, respectively. As reported in Reference [67], the emission of these CDs is enhanced by the interaction with $Zn^{2+}$ ions, whereas the CD emission is quenched by Co^2+^, as shown in Figure S7. The energy position of the excited state of CDs is thereby between those of the acceptor level of these two ions, which can be quantified by their redox potentials. In terms of energy, the redox potentials of zinc and cobalt correspond to the energies E_$Zn^{2+}$_ = −3.8 eV and E_$Co^{2+}$_ = −4.2 eV, thus placing in this range the energy level of CD surface states E_CDs_. Finally, the valence band of CD core can be placed about 2 eV below the surface state, considering the lack of light absorption at energy below this threshold.

Therefore, since E_CDs_ −φ_Gr_ > 0, the surface electron of photoexcited CDs can migrate to the unoccupied states in the conduction band of Gr, hence, the PET between the two nanomaterials spontaneously occurs. Finally, the transferred electron is expected to undergo an ultrafast relaxation towards lower energy states, that is, the valence band states of Gr [68,69]. As PET causes an increase of the occupancy of the states just above the Fermi level, it is equivalent to an n-doping of graphene dynamically induced by photo-excitation of CDs. On the other hand, when CDs are in their electronic ground state (as in the case of measurement performed at 1.96 eV, when the light absorption of CDs is prevented), PET towards Gr cannot be activated, and no modification of Fermi energy is evidenced in p-Gr Raman signal. The PET from CDs to Gr dynamically induced by the laser excitation is thereby evidenced by (1) the increase of Fermi energy evaluated by the evolution of Gr Raman signal which reveals an n-doping which reduces the previously induced p-doping; (2) the inhibition of CD PL, since the separation of the excitonic pair interrupts the photocycle.

Some considerations originate from this model. As commonly found in literature, the charges accumulated in graphene are expected to be ceded to the surrounding environment. Therefore, the n-doping, due to PET from CDs, is expected to fade once the laser irradiation is stopped [28,29]. Considering the very strong PL quenching observed for CDs, it is likely that the time scale of the electron transfer (ET) responsible for it is orders of magnitude faster than the native lifetime (a few nanoseconds). As a matter of fact, studies of ET from CDs to model metal ions have found ET to proceed on picosecond and subpicosecond time scales [67]. Further dedicated studies would be needed to directly address such anticipated ultrafast dynamics. According to the used laser power, an increase of Fermi energy at least equal to 0.2 eV is estimated by the Raman analysis, corresponding to up to 5 × 10^12^ cm^−2^ transferred electrons for the nominal power we used. However, a modulation of such value is expected upon varying laser power.

## 4. Conclusions

In summary, the interaction between CDs and single-layer graphene has been investigated by designing a novel experimental strategy which allows us to simultaneously probe the electronic properties of both materials. Our data provide strong evidence of PET from CDs to graphene. In particular, from a structural perspective, the deposition of CDs induces only minor modification in Gr strain, hence, indicating a preservation of Gr structure. Besides, CDs deposited on both undoped and p-doped Gr feature a reduced emission efficiency, highlighting the opening of a new channel for the relaxation of photoexcited CDs. The Raman study demonstrates that Gr features a different density of charge carriers only after the photoexcitation of CDs deposited on it, especially for CDs/p-Gr, where the effect is more evident. These changes are interpreted in terms of a PET from photoexcited CDs to Gr allowed by their close contact and the favorable energy alignment of CDs and Gr levels. The reported results suggest this specific solid-phase CD-Gr nanocomposite as a promising nanosystem for light conversion applications, such as light harvesting and optoelectronic devices, where usual liquid-phase graphene-nanoparticle composite materials are precluded.

## Supplementary Materials

The following are available online at https://www.mdpi.com/2079-4991/10/3/528/s1.
