# Supplementary material for: Dynamic Modification of Fermi Energy in Single-Layer Graphene by Photoinduced Electron Transfer from Carbon Dots
Source: Nanomaterials (Basel). 2020 Mar 15;10(3):528. doi: 10.3390/nano10030528 (PMC7153610; doi:10.3390/nano10030528)
Supplement: Supplementary file 1 [file nanomaterials-10-00528-s001.zip › suppinfo.pdf]

Supporting Information

Dynamic Modification of Fermi Energy in Single Layer Graphene by Photoinduced Electron Transfer from Carbon Dots

A. Armano[a,b], G. Buscarino[a,c,d], F. Messina[a,c], A. Sciortino[a] M. Cannas[a], F. M. Gelardi[a], F. Giannazzo[d], E. Schilirò[d], and S. Agnello[a,c,d,*]

a Università degli Studi di Palermo, Dipartimento di Fisica e Chimica,Via Archirafi 36, 90123 Palermo, Italia

b Università degli Studi di Catania, Dipartimento di Fisica e Astronomia, Via Santa Sofia 64, 95123 Catania, Italia

c Università degli Studi di Palermo, ATeN Center, Viale delle Scienze, Edificio 18, 90128 Palermo, Italia

d Consiglio Nazionale delle Ricerche—Istituto per la Microelettronica e Microsistemi, Strada VIII 5, 95121 Catania, Italia

* Corresponding Author

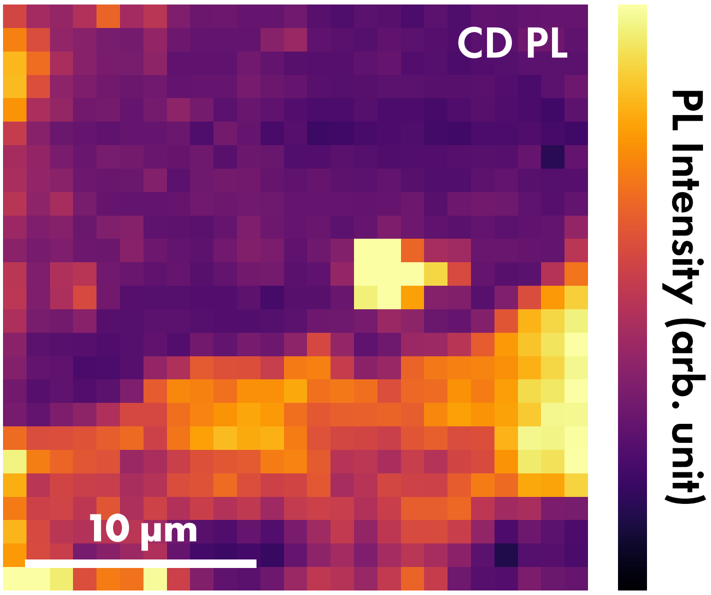

Figure S1: μ-PL Mapping shown in Figure 5b reported without pixel interpolation.

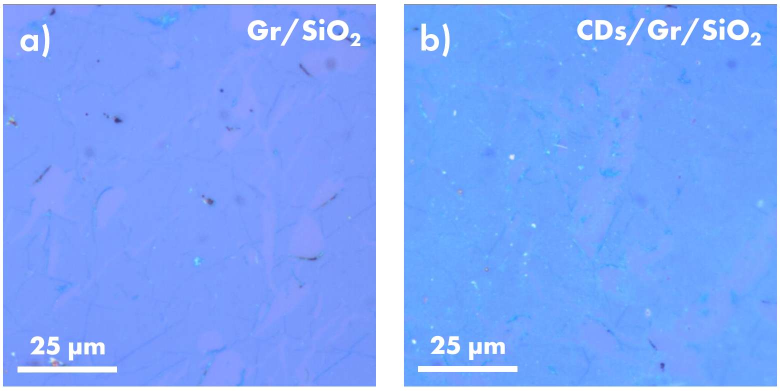

Figure S2: Optical images shown in Figure 3a,b reported without color curves correction.

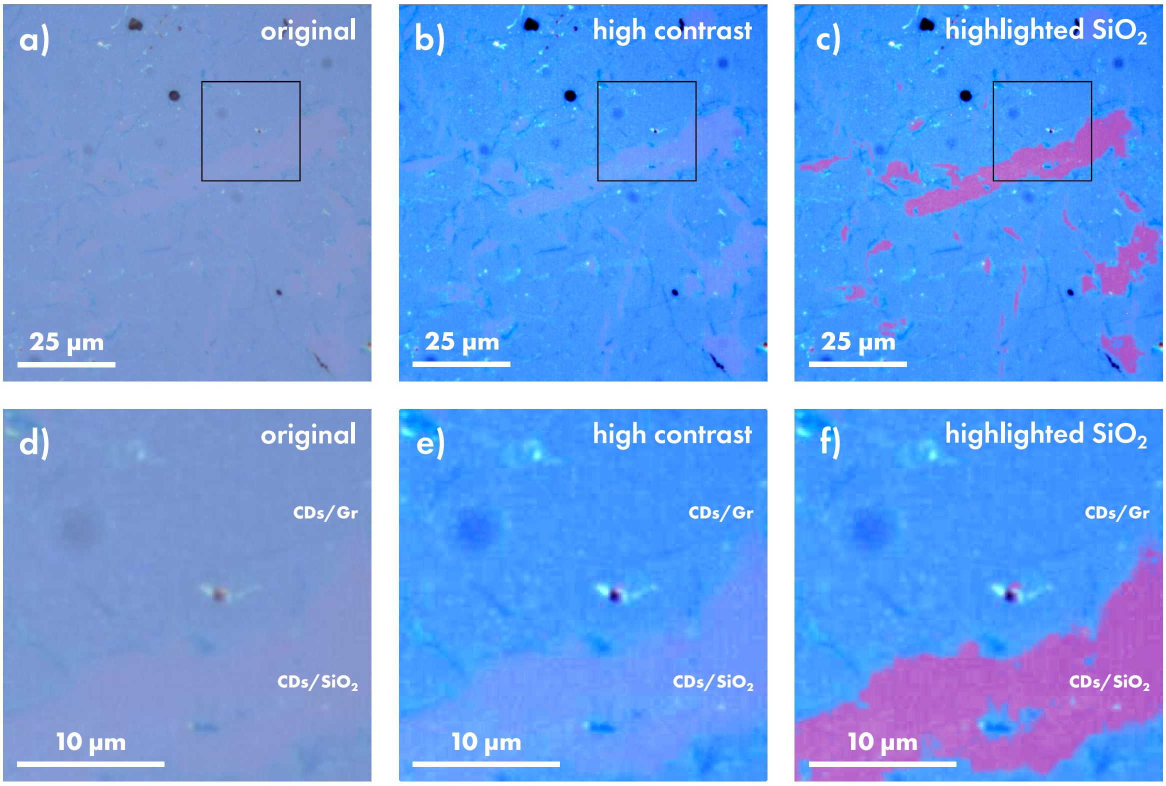

Figure S3: Optical images shown in Figure 5a reported in original and cropped version **(a,d)** before and **(b,e)** after color curves correction, and **(c,f)** with highlighted surface SiO_2 regions. The selected region is pointed out in panels **(a,b,c)** by black squares border.

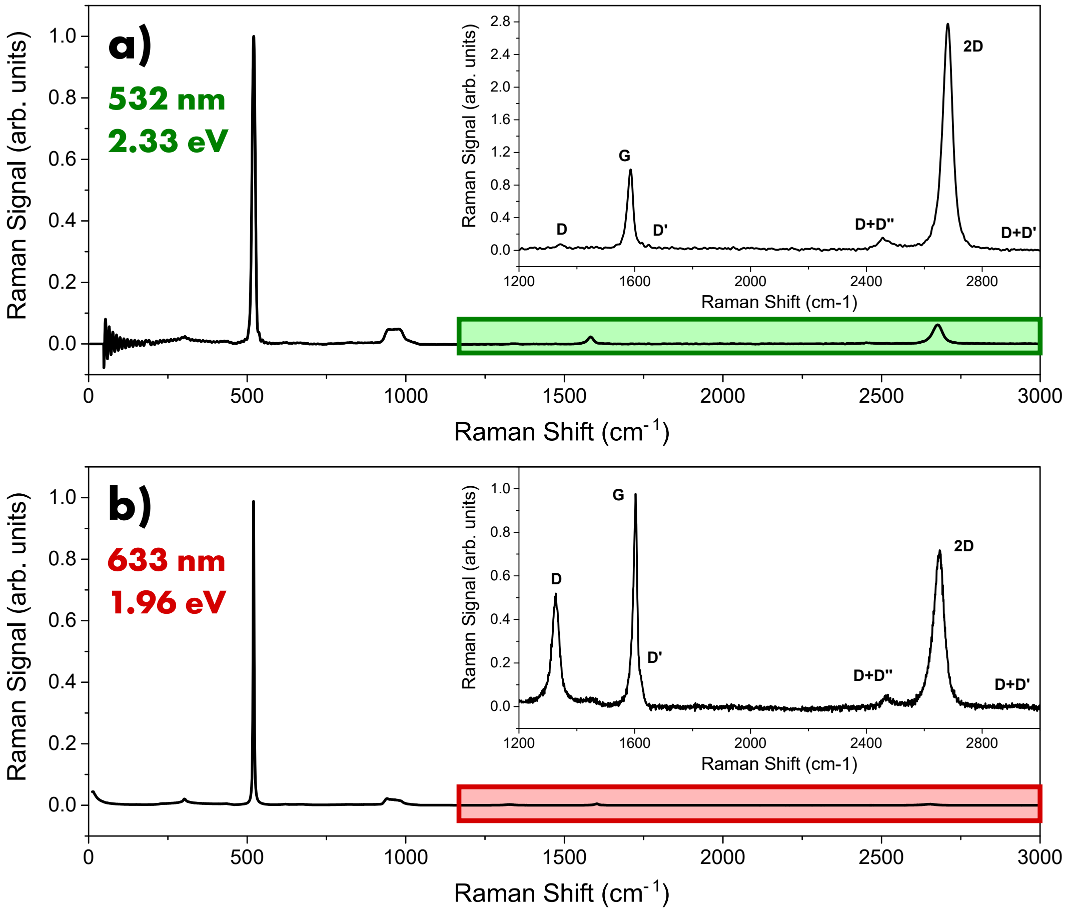

Figure S4: Raman spectrum of undoped graphene acquired by using laser at **(a)** 532 nm (2.33 eV) and **(b)** 633 nm (1.96 eV). In both panels, insets show the spectral range between 1200–3000 cm^{-1} containing the main bands of graphene.

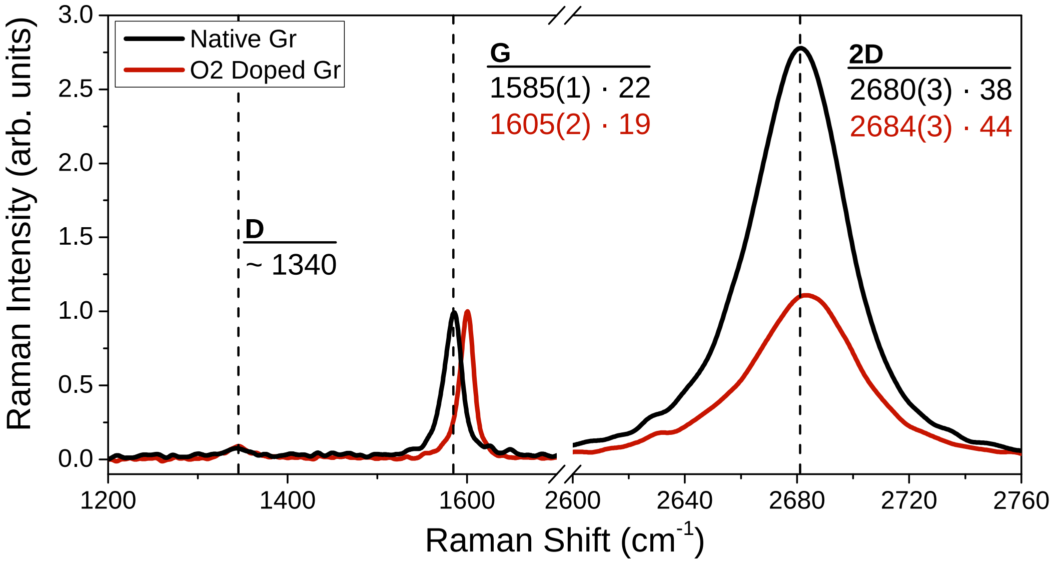

Figure S5: Raman spectra of undoped (black) and p-doped graphene (red) acquired by using laser at 532 nm. The mean value of peak position and FWHM of main bands are reported. Adapted from Ref. [41]

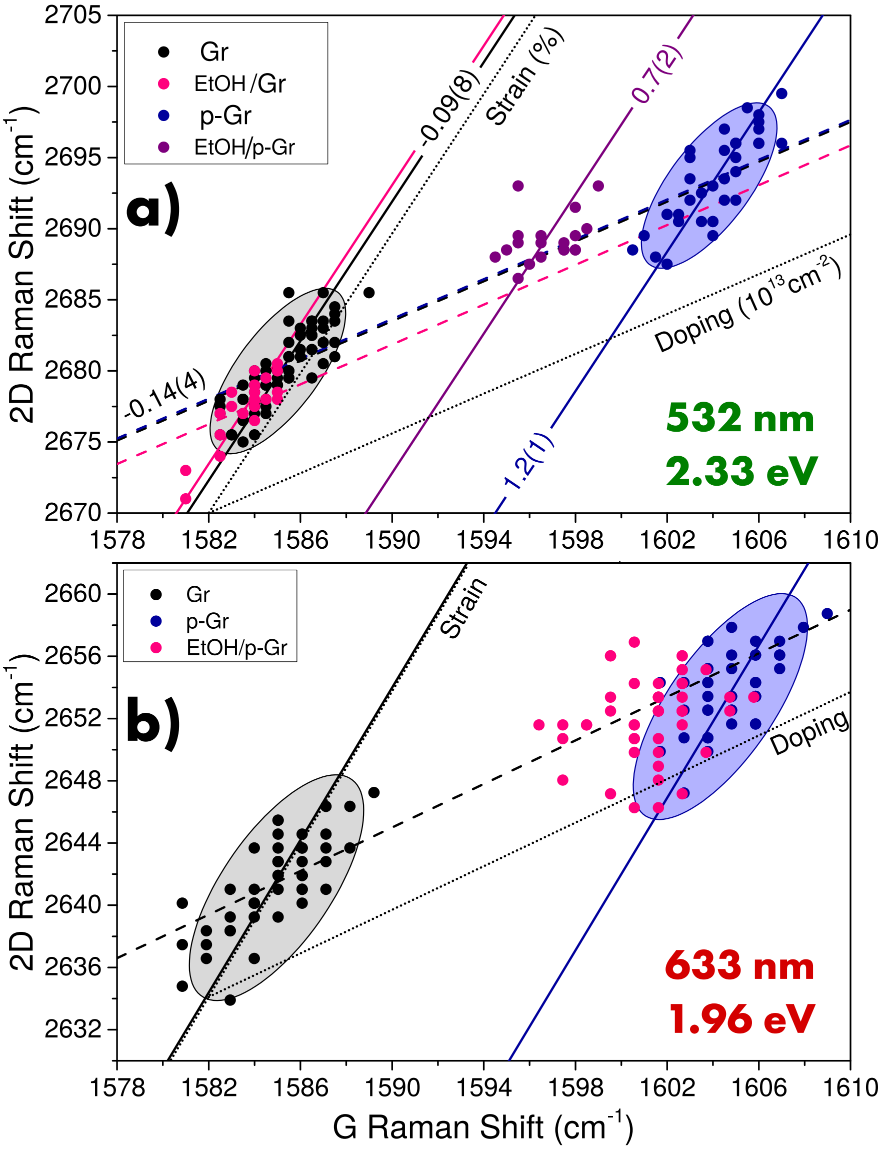

Figure S6: G-2D correlation map at **(a)** 532 nm and **(b)** 633 nm laser excitation of undoped and p-doped Gr (reported from Ref. [41] for 532 nm only), undoped and p-doped graphene previously wetted in ethanol. Doping and strain axes are marked by dotted lines and reference levels are pointed out by countinuous and dashed lines, respectively.

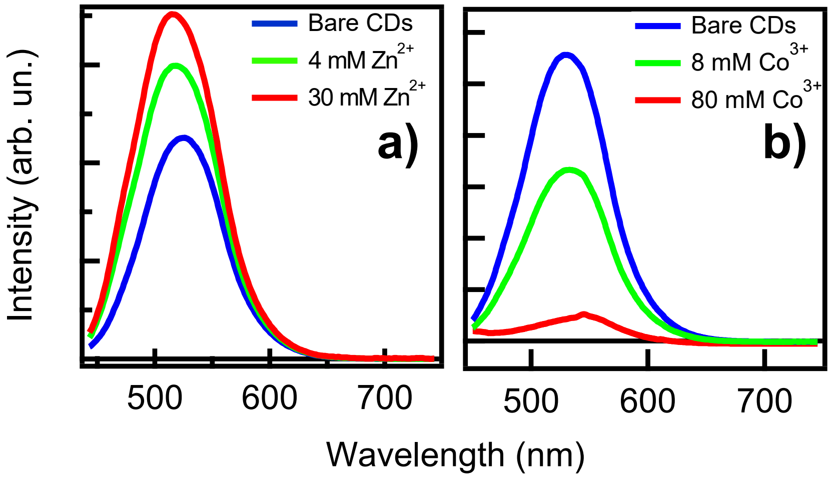

Figure S7: Emission spectra of bare CDs (blue) with **(a)** zinc, and **(b)** cobalt ions in solution. Different concentrations of ions are compared (green, red).
