# Supplementary figures and images for: Dynamic Modification of Fermi Energy in Single-Layer Graphene by Photoinduced Electron Transfer from Carbon Dots
Source: Nanomaterials (Basel). 2020 Mar 15;10(3):528. doi: 10.3390/nano10030528 (PMC7153610; doi:10.3390/nano10030528)

**PL Intensity (arb. unit)**

**CD PL**

**10  $\mu\text{m}$**

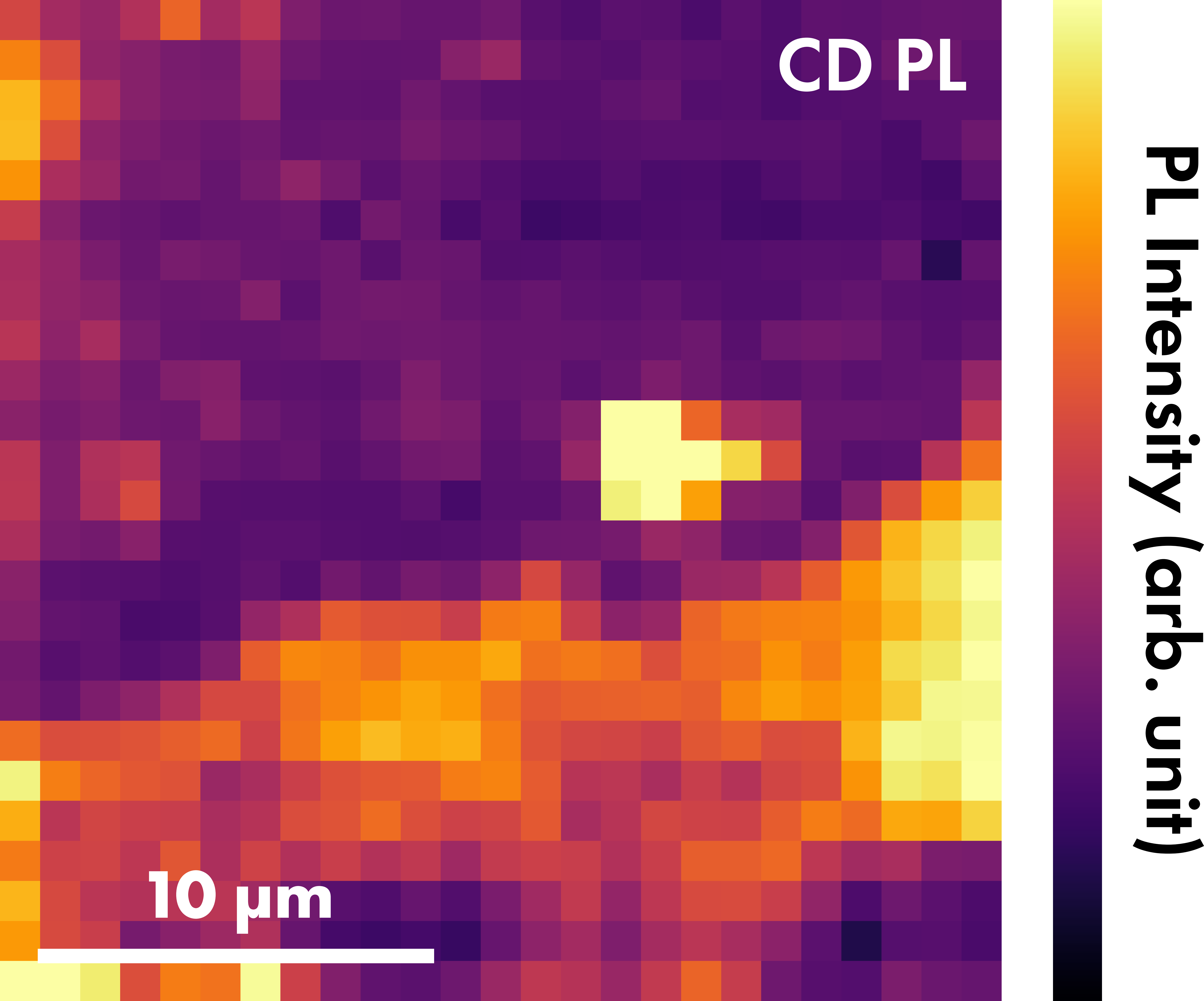

Supplement: Supplementary file 1 [file nanomaterials-10-00528-s001.zip › Sup_1.pdf]

**a)**

**Gr/SiO<sub>2</sub>**

**25 μm**

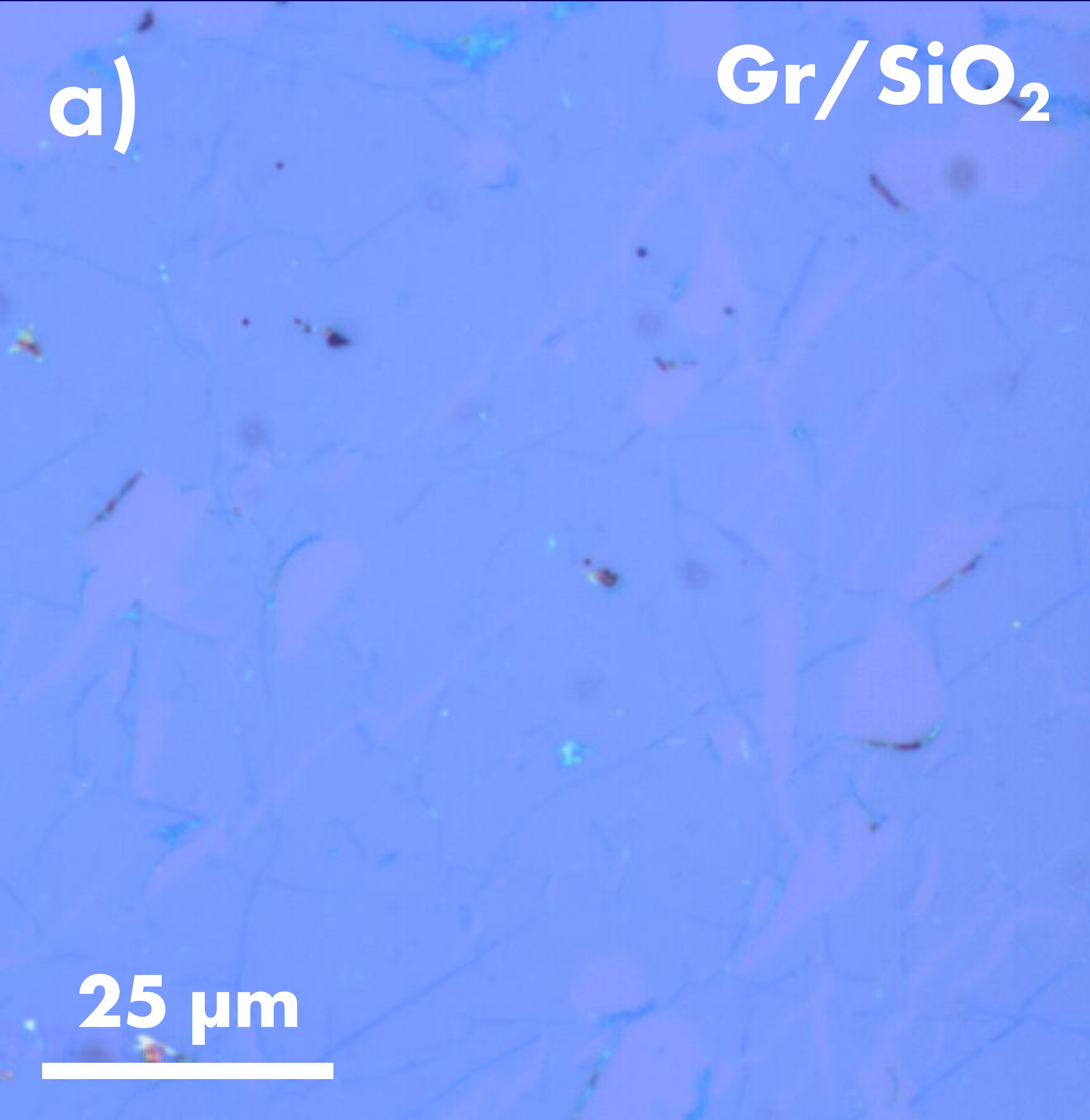

**b)**

**CDs/Gr/SiO<sub>2</sub>**

**25 μm**

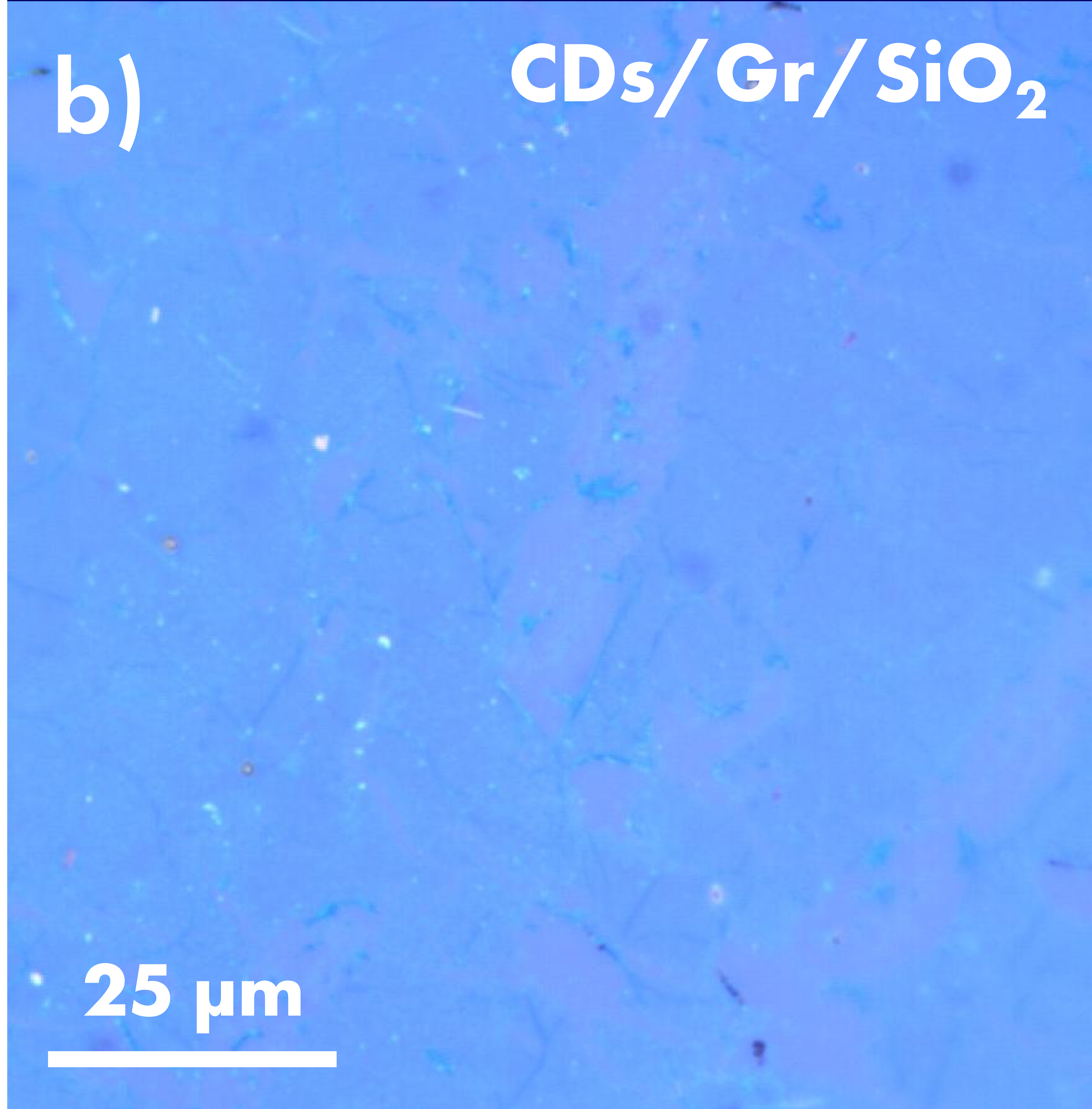

Supplement: Supplementary file 1 [file nanomaterials-10-00528-s001.zip › Sup_2.pdf]

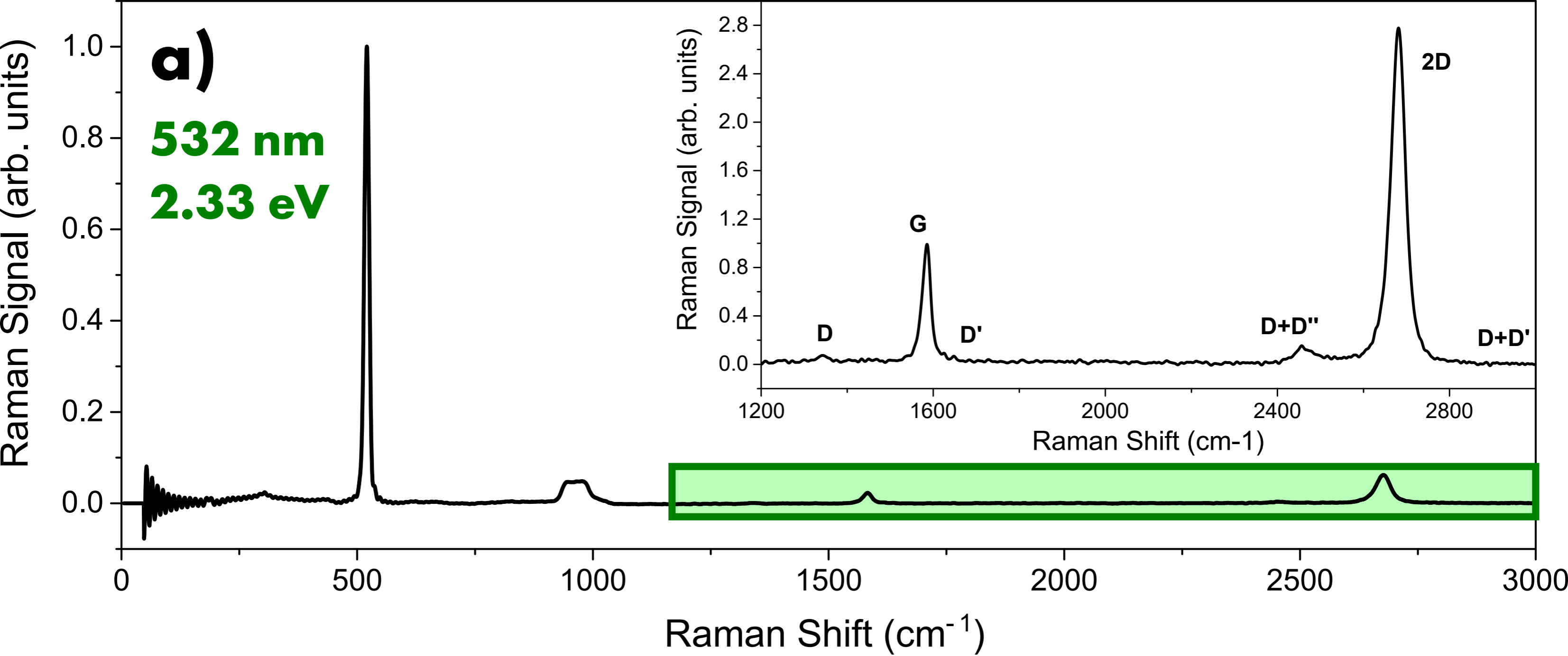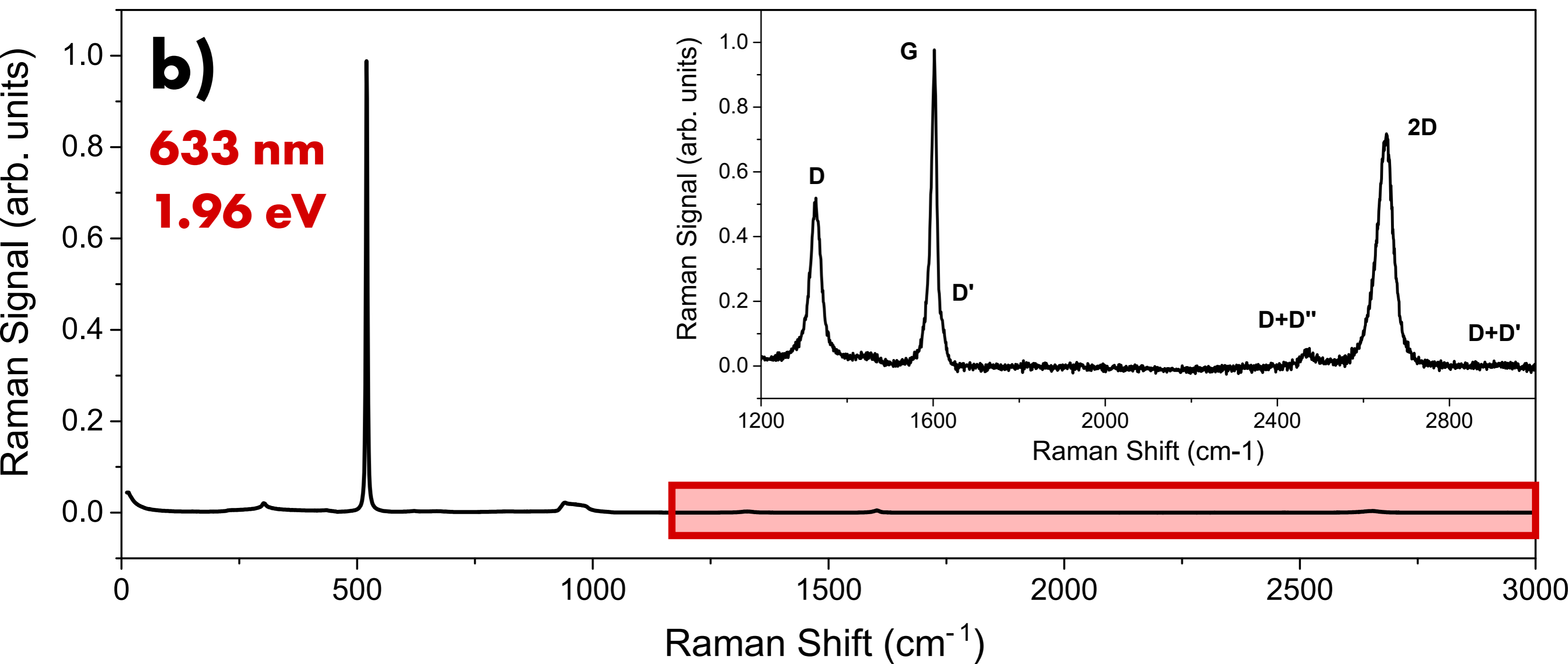

Supplement: Supplementary file 1 [file nanomaterials-10-00528-s001.zip › Sup_4.pdf]

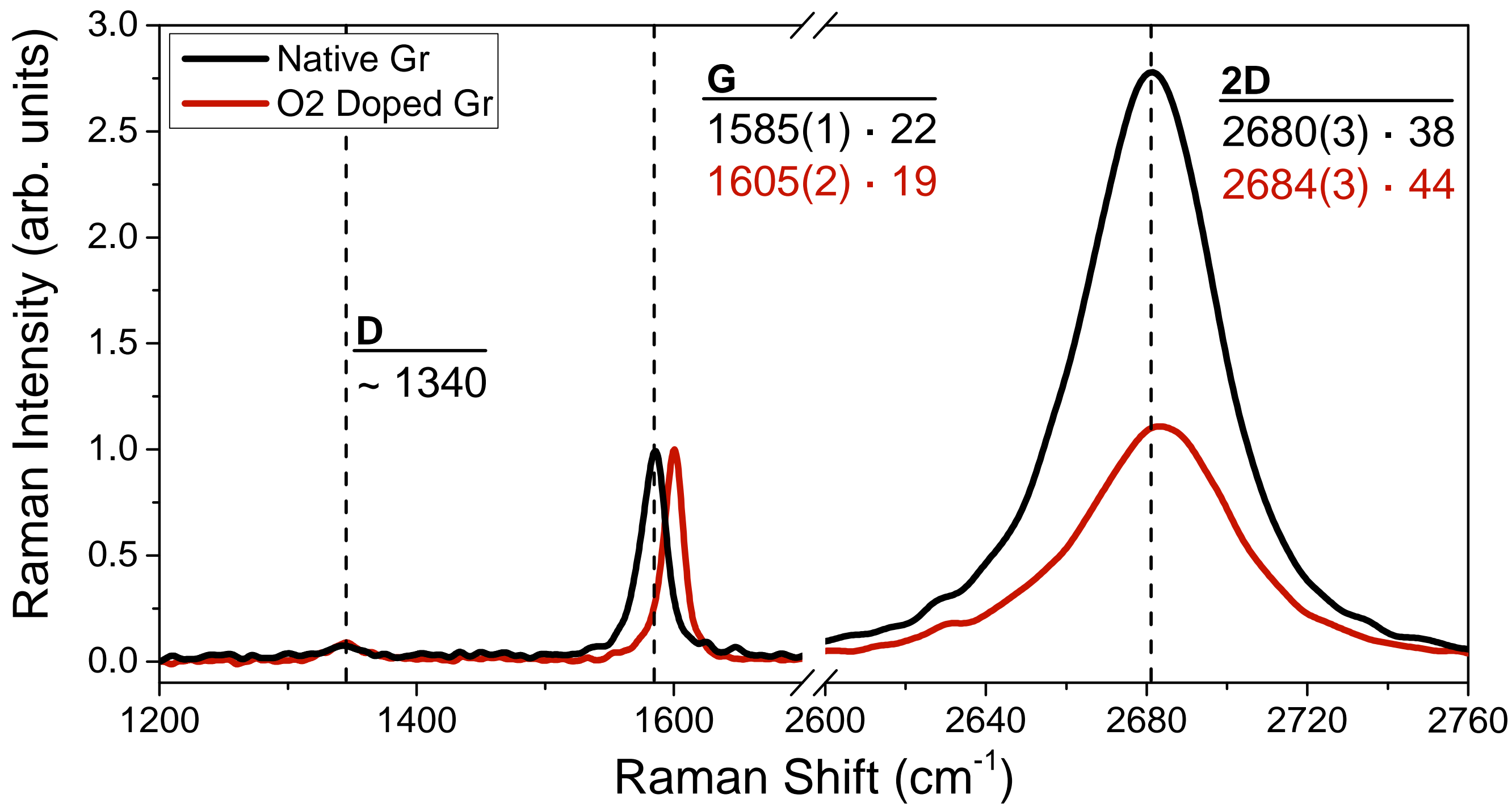

Supplement: Supplementary file 1 [file nanomaterials-10-00528-s001.zip › Sup_5.pdf]

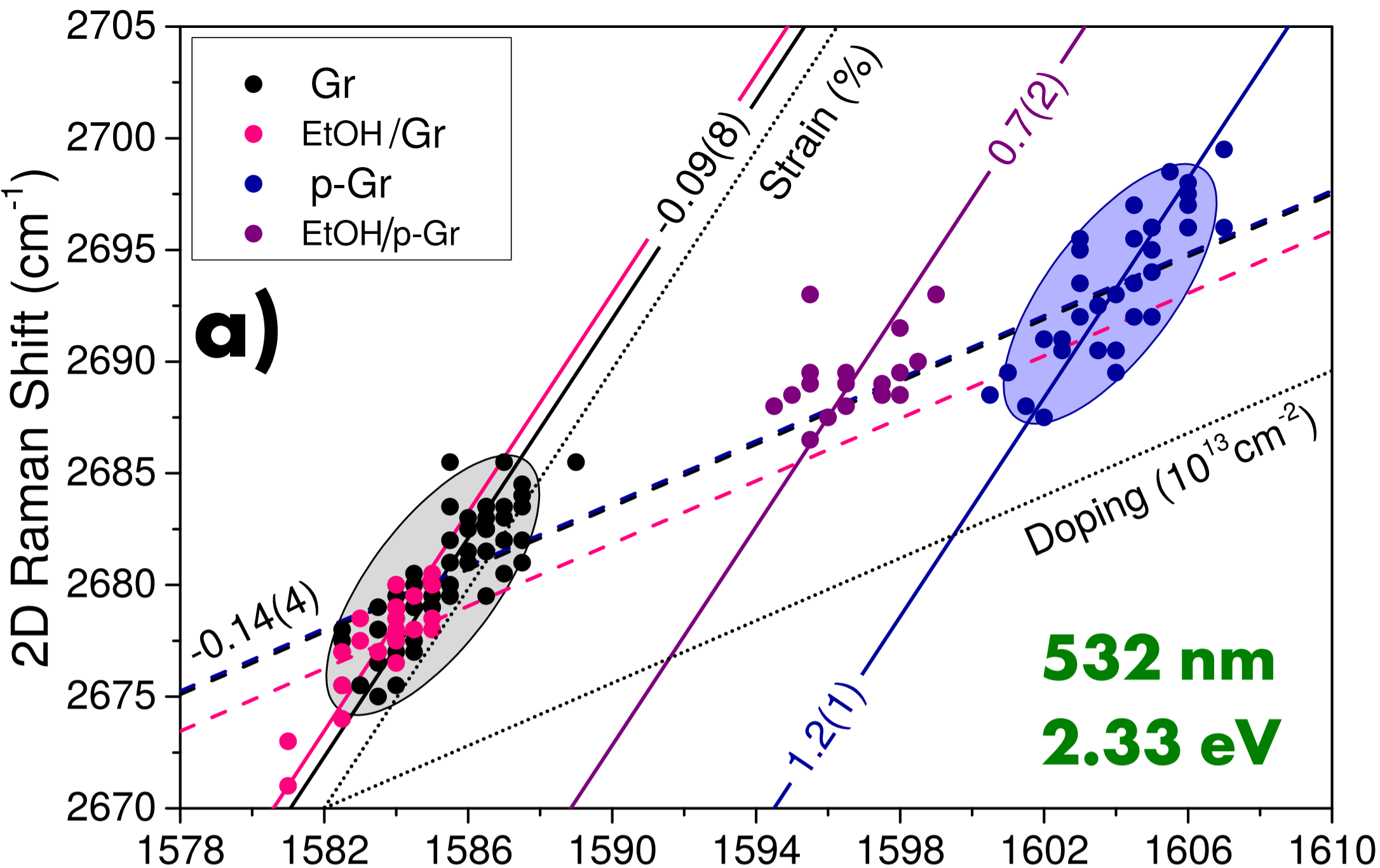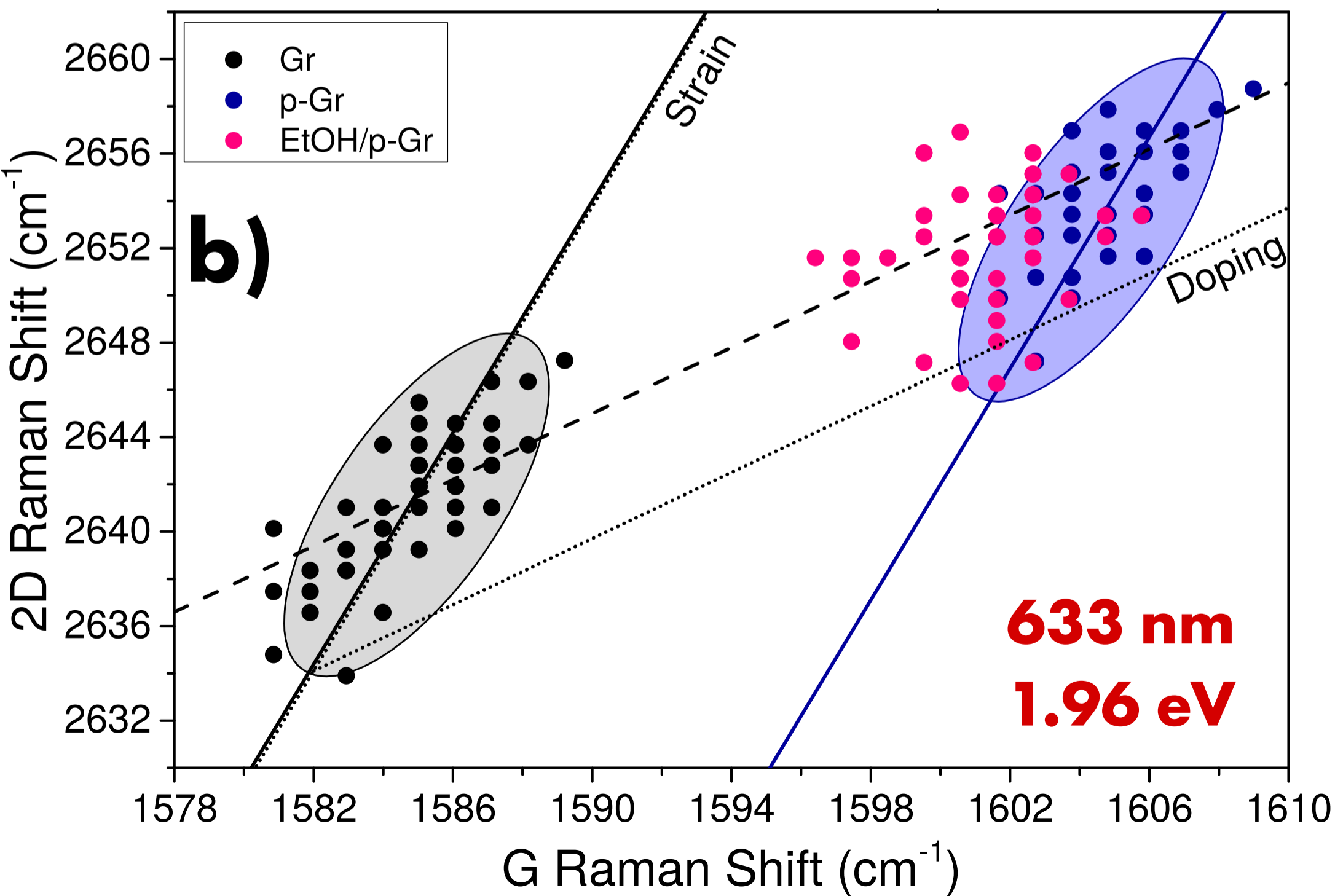

Supplement: Supplementary file 1 [file nanomaterials-10-00528-s001.zip › Sup_6.pdf]

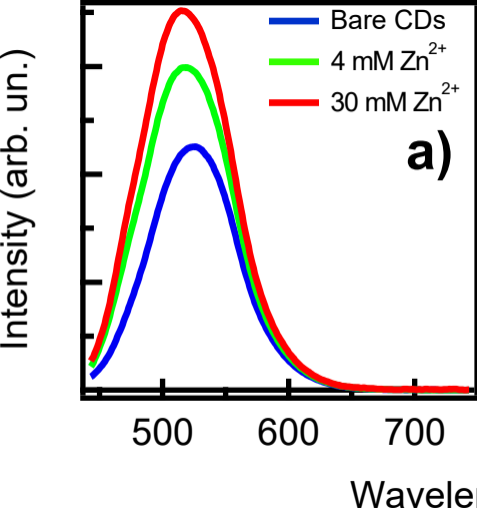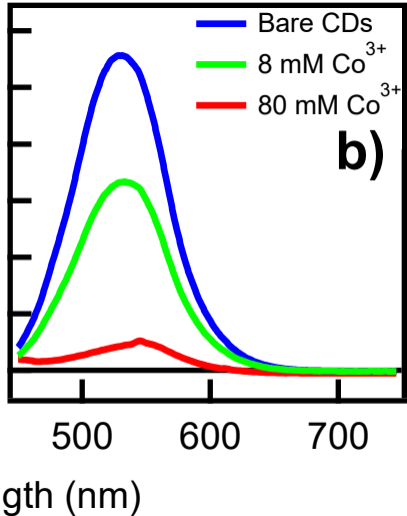

Supplement: Supplementary file 1 [file nanomaterials-10-00528-s001.zip › Sup_7.pdf]
