# Supplementary material for: Dynamic Modification of Fermi Energy in Single-Layer Graphene by Photoinduced Electron Transfer from Carbon Dots
Source: Nanomaterials (Basel). 2020 Mar 15;10(3):528. doi: 10.3390/nano10030528 (PMC7153610; doi:10.3390/nano10030528)
Supplement: Supplementary file 1 [file Sup_3.pdf › Sup_3.pdf]

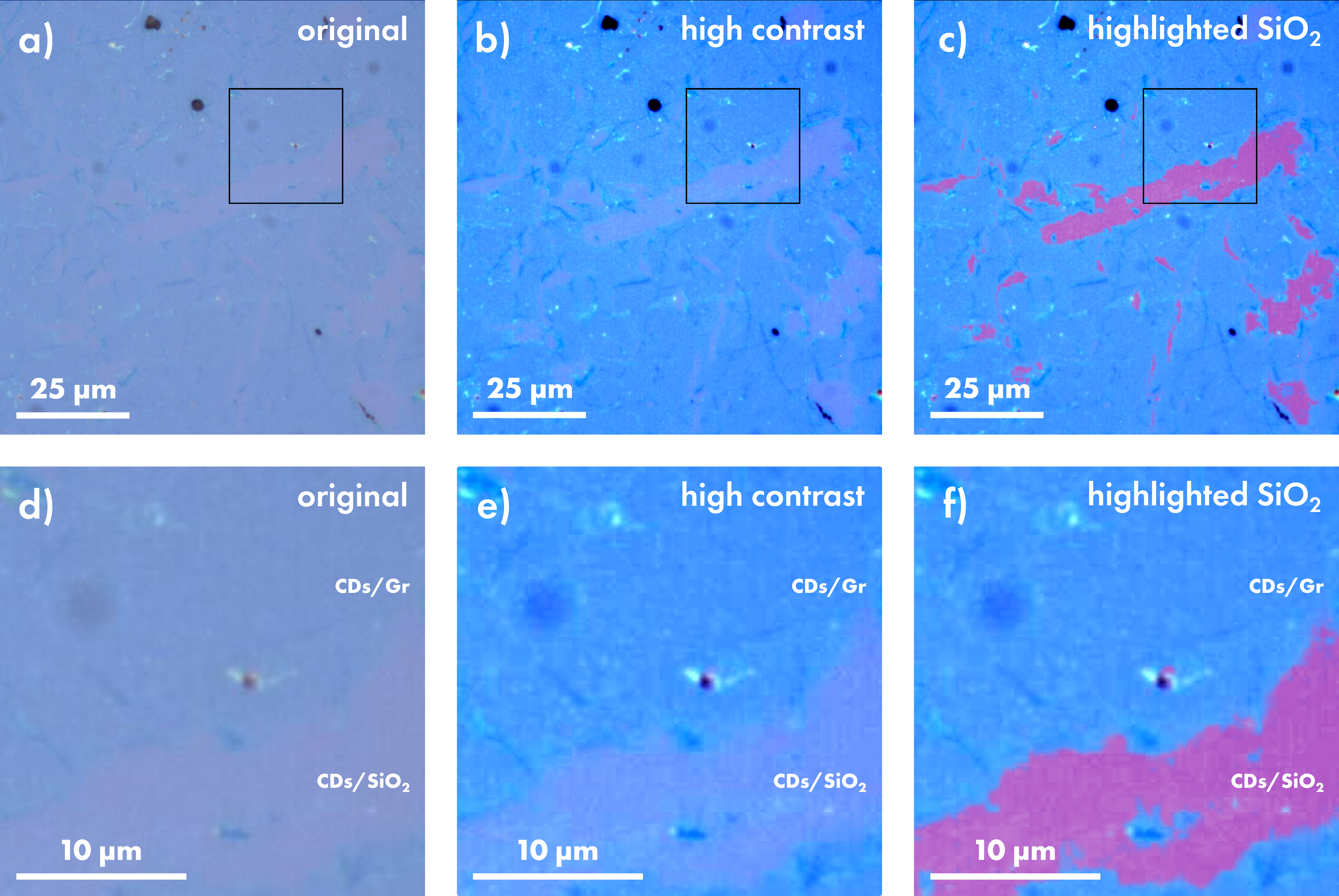
a)
original
25 µm
b)
high contrast
25 µm
c)
highlighted SiO_2
25 µm
d)
original
CDs/Gr
CDs/SiO_2
10 µm
e)
high contrast
CDs/Gr
CDs/SiO_2
10 µm
f)
highlighted SiO_2
CDs/Gr
CDs/SiO_2
10 µm
